# An Unusual Case of Myocarditis, Left Ventricular Thrombus, and Embolic Stroke Caused by Mycoplasma pneumoniae

**DOI:** 10.7759/cureus.14170

**Published:** 2021-03-29

**Authors:** Mansi Oberoi, Raksha Kulkarni, Tony Oliver

**Affiliations:** 1 Internal Medicine, University of South Dakota Sanford School of Medicine, Sioux Falls, USA

**Keywords:** myocarditis, thrombus, stroke, mycoplasma pnemoniae

## Abstract

*Mycoplasma pneumoniae *is a common cause of community-acquired pneumonia, but it can affect other parts of the body. Due to the varied presentation and lack of readily available specific diagnostic tools, diagnosis is often challenging, which may lead to delay in the treatment and unfavorable outcomes. We describe such a unique case of myocarditis caused by *Mycoplasma pneumoniae* complicated by left ventricular thrombus and an embolic stroke without the presence of pneumonia. There is a paucity of data regarding *Mycoplasma pneumoniae* myocarditis and stroke in the absence of pulmonary symptoms especially in adults, calling for further studies for early diagnosis and management.

## Introduction

*Mycoplasma pneumoniae* is a known causative agent of community-acquired pneumonia and has been reported in 10%-40% of community-acquired pneumonia cases, with an overall mortality of around 30% [1]. Besides lung involvement, patients may develop several extra-pulmonary manifestations including cardiovascular, dermatological, gastrointestinal, hematological, and neurological involvement [2]. Thrombosis, especially cardiac thrombosis associated with M. pneumonia, is extremely rare [3,4]. We present such an unusual case of *M. pneumoniae* infection presenting with left ventricular thrombus and an embolic stroke in an adult.

## Case presentation

A previously healthy 37-year-old male without any past medical history presented with a three-week history of sore throat, exertional dyspnea, orthopnea, and bilateral lower extremity swelling. He also reported having chest pain associated with non-productive cough and a few episodes of foamy green stools. The review of other systems was unremarkable. He completed a course of amoxicillin one week back for presumed pharyngitis without any significant improvement.

On physical examination, he was hemodynamically stable, afebrile with a heart rate of 103 beats/min, respiratory rate of 40/min, and oxygen saturation of 95% on room air. He was oriented to time, place, and person, with no focal neurological deficits. There were diminished bibasilar breath sounds without any wheeze or crackles along with an apical systolic 2/6 murmur and bilateral lower extremity pitting edema.

**Figure 1:**
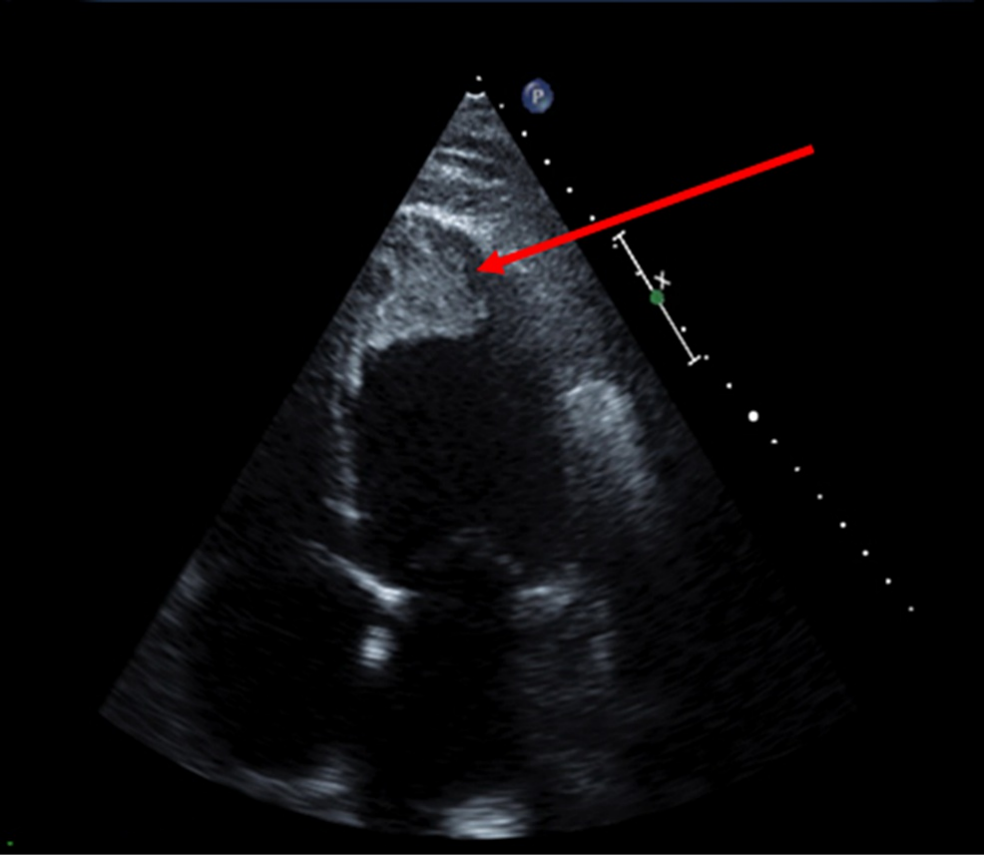
Transthoracic echocardiogram showing apical thrombus in the dilated left ventricle

Laboratory workup revealed elevated troponin at 0.39 ng/mL (normal: 0-0.03 ng/mL) and brain natriuretic peptide (BNP) at 2,500 pg/mL (normal: 0-100 pg/mL). Rapid streptococcal group A test was negative. Electrocardiogram (EKG) did not show any acute ischemic changes and no evidence of acute myocardial infarction. Chest X-ray revealed cardiomegaly with no evidence of infiltrates suggestive of pneumonia or edema. Transthoracic echocardiography (TTE) showed severe left ventricular dilatation with a reduced ejection fraction of 10%. An apical left ventricular thrombus (Figure 1) was noted, with moderate-to-severe mitral regurgitation along with small pericardial effusion without tamponade physiology.

The patient’s symptoms of exertional dyspnea, orthopnea, chest pain, and bilateral lower extremity edema, along with elevated BNP and slightly elevated troponin levels, favored the diagnosis of acute onset myocarditis and heart failure. The patient was treated with diuretics (furosemide) and started on American College of Cardiology (ACC)/American Heart Association (AHA) guideline-directed medical therapy for heart failure, including angiotensin convertase enzyme inhibitor (lisinopril 5 mg) and beta-blocker (metoprolol succinate 25 mg) once he was euvolemic. Heparin infusion was initiated for his left ventricular thrombus. Acute myocarditis and heart failure in a relatively healthy young male prompted an infectious and autoimmune workup including Epstein-Barr virus (EBV), cytomegalovirus (CMV), human immunodeficiency virus (HIV), coxsackievirus, *M. pneumoniae*, serum protein electrophoresis, antinuclear antibody, and amyloidosis. The results came back positive for mycoplasma IgM, following which he was started on azithromycin.

**Figure 2:**
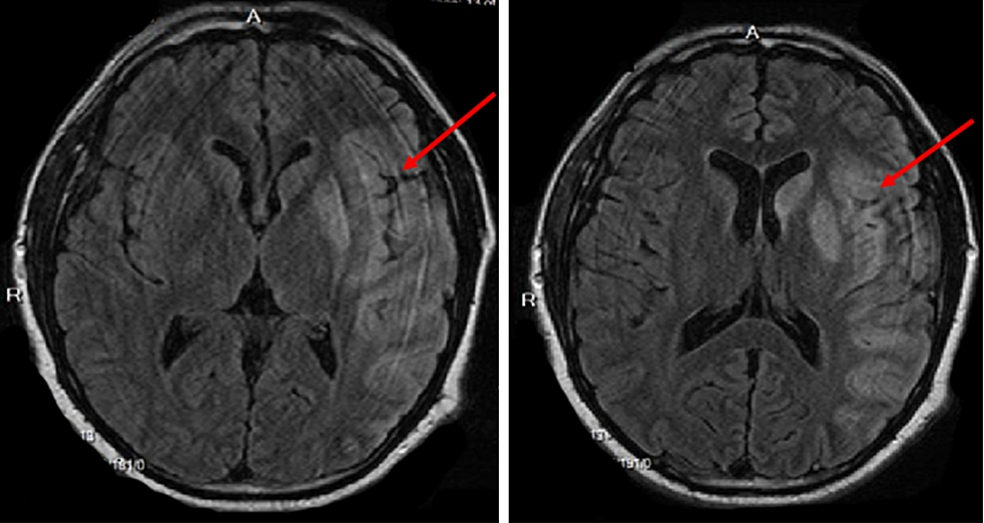
Magnetic resonance imaging (MRI) of the brain showing hyperintense areas in the left frontotemporal and parietal lobes including globus pallidus

**Figure 3:**
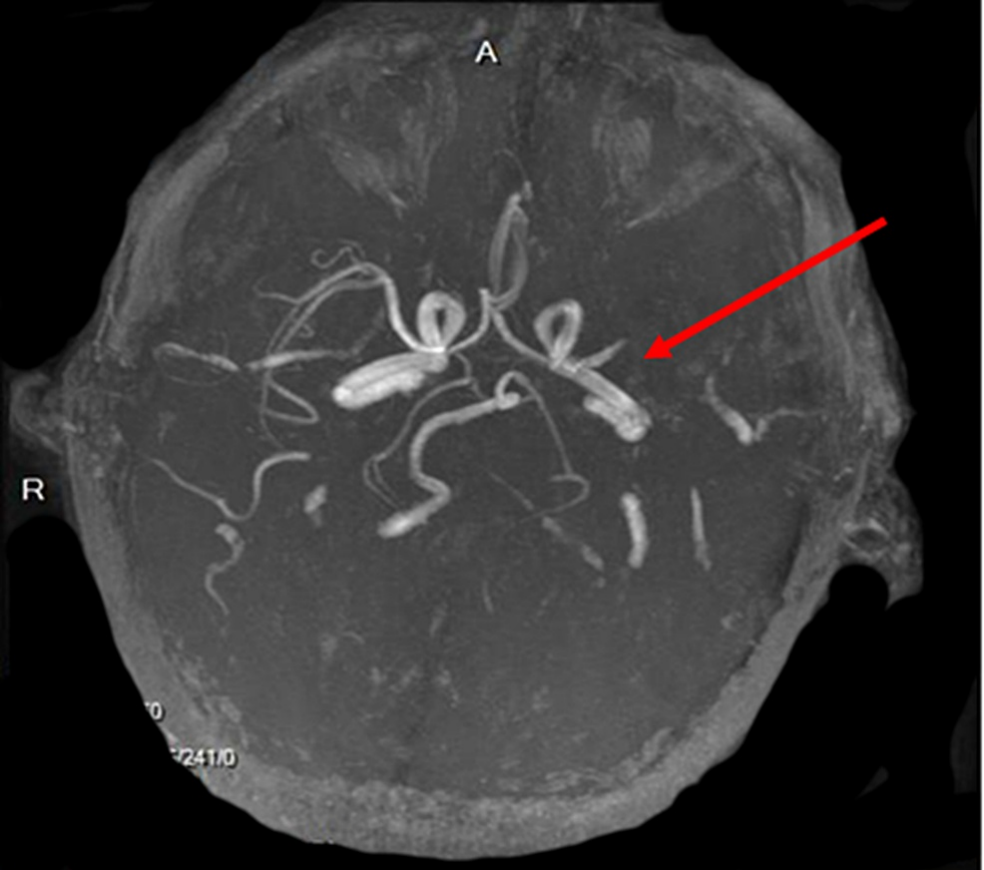
Magnetic resonance angiography of the head showing left middle cerebral artery proximal M1 segment occlusion (red arrow)

On the second day of hospitalization, he developed an acute ischemic stroke with right-sided hemiplegia and global aphasia. Magnetic resonance imaging (MRI) of the brain revealed an acute infarction of a large area of left middle cerebral artery (MCA) territory and of multiple small areas in both cerebral hemispheres consistent with an embolic pattern, likely due to the dislodgement of the left ventricular thrombus (Figure 2). MR angiogram head demonstrated left MCA proximal M1 segment occlusion (Figure 3). Recombinant tissue plasminogen activator could not be administered due to his elevated INR (international normalized ratio) on admission for an unknown cause. He was intubated and placed on therapeutic hypothermia. Later, he developed cardiogenic shock and unfortunately died on the eighth day of admission.

## Discussion

*Mycoplasma pneumoniae* infections remain highly underdiagnosed as the majority of patients remain asymptomatic. Approximately 50% of patients present with mild upper respiratory tract symptoms including sore throat, fever, cough, and malaise, and, around 3%-10% of patients develop pneumonia [5]. However, some may develop more severe manifestations including acute respiratory distress syndrome and diffuse alveolar hemorrhage [1].

Several extrapulmonary manifestations are associated with *M. pneumoniae*, which include cardiovascular (pericarditis, myocarditis, endocarditis), dermatological (erythema multiforme, urticaria, Stevens-Johnson syndrome), hematological (hemolytic anemia, infectious mononucleosis), musculoskeletal (arthritis), and neurological (encephalitis, myelitis, aseptic meningitis) involvement, but these are uncommon in the absence of pneumonia [2,6]. Thrombosis associated with *M. pneumoniae* is extremely rare, especially among adults [3]. The varied and indistinct symptom presentation of *M. pneumoniae* infection makes it difficult to distinguish it from respiratory tract infections caused by other viruses and atypical bacteria. The presumed benign nature of the infection, lack of diagnostic tests with sufficient sensitivity and specificity, co-existing diseases, and infections mimicking *M. pneumoniae* also make the diagnosis challenging [1].

While extrapulmonary manifestations of *M. pneumoniae* are well known, there is no consensus on its pathophysiology. Various theories that have been postulated regarding the etiology of extrapulmonary manifestations include direct invasion by the microorganism, autoimmune response, or vascular occlusion leading to vasculitis or thrombosis in the presence or absence of a systemic hypercoagulable state induced by the bacterium [2,7]. The autoimmune mechanism is considered most likely in our case due to a lag period between symptom onset and development of extrapulmonary manifestations. Thromboses, including cardiac thrombi, are seen more commonly in children unlike our case [8].

Among the infectious causes of myocarditis, viruses are the most common pathogens including enteroviruses particularly coxsackievirus, adenovirus, parvovirus B19, human herpesvirus 6, hepatitis C, EBV, CMV, and HIV. The true incidence of viral myocarditis is unknown. These infections are generally present in children and are often associated with rash and nasal/throat symptoms. Among bacterial causes, *Corynebacterium diphtheriae* and *Staphylococcus aureus* are frequent pathogens. However, *M. pneumoniae* should also be considered amongst the differentials of pericarditis and myopericarditis [9]. Serology, cultures, and polymerase chain reaction (PCR) of blood and cardiac tissue are often used for the detection of the pathogen.

The mainstay for the treatment of *M. pneumoniae* infection remains the use of antibiotics for both pulmonary and extrapulmonary manifestations. All extrapulmonary manifestations must be treated with antibiotics as direct invasion of the organisms cannot be ruled out [1]. The majority of cases are treated with macrolides, but in areas with high resistance to macrolides, doxycycline or fluoroquinolones may be considered [10]. Thrombi are often successfully treated with anticoagulant therapy [8]. There are conflicting reports regarding the benefits of steroids, plasmapheresis, and intravenous immunoglobulin therapy [1].

## Conclusions

*Mycoplasma pneumoniae* can cause a myriad of extrapulmonary manifestations even in the absence of pulmonary symptoms. Some of these manifestations are not directly related to the infectious process but are autoimmune or vascular in nature. A high degree of clinical suspicion should be entertained when it comes to *M. pneumoniae* infections as they present with subtle signs and symptoms that may lead to serious pulmonary and extrapulmonary manifestations. Macrolides are the drugs of choice even for extrapulmonary manifestations.
